# Minimally Invasive Approach for Excision of Dorsolumbar Spine Osteoid Osteomas using 3D Imaging and Stealth Navigation: A Report of Two Cases

**DOI:** 10.5704/MOJ.2207.017

**Published:** 2022-07

**Authors:** RT Sakhrekar, VB Peshattiwar, P Rathi, M Gawande

**Affiliations:** Department of Spine, Kokilaben Dhirubhai Ambani Hospital and Medical Research Institute, Mumbai, India

**Keywords:** osteoid osteoma, excision, minimally invasive approach, computer navigation, O-Arm

## Abstract

Advantages of three-dimensional (3D) computed tomography-based navigation system has recently been used for safe, accurate, reliable spinal tumour excision. This study presents two cases, a 33-year-old male and a 17-year-old male presented in the clinic with mid-back and low back ache, respectively with change in posture. Radiology suggested an osteoid osteoma. Accurate localisation and complete extirpation of the lesion were performed using a translaminar approach with O-arm Navigation. After follow-up of one year, both did not complain of back pain or radiation, scoliosis had improved in both cases and their VAS was 0 and ODI was 0%. 3D navigation with the O-arm system provided an easy and accurate localisation of the lesion, reducing the risk of instability subsequently and avoiding instrumented stabilisation. This technique also provided for histopathological confirmation of the diagnosis.

## Introduction

Osteoid osteoma is a primary benign bone lesion that commonly occur in the second decade of life, but also varies from the first to fourth decade with a male to female predominance of 2:1. The clinical symptom reported is night pain, caused by nidus induced prostaglandin/prostacyclin production. Patients also present with painful scoliosis due to soft tissue and muscle irritation, leading to asymmetrical spasm. Surgical excision is the definitive method of treatment for patients unresponsive to nonsteroidal anti-inflammatory drugs like aspirin^1^. Over the decades, surgical techniques of the spine have evolved, and minimally invasive procedures are now utilised for excision of osteoid osteoma with high precision. We report two cases of osteoid osteoma of spine managed with surgical excision with O-arm guided navigation. Case 1 had osteoid osteoma at superior articular process of the D11 vertebra while Case 2 had osteoid osteoma of the L4 right pars interarticularis, both treated with surgical excision with O-arm guided navigation.

## Case Report

### Case 1

**Fig 1:**
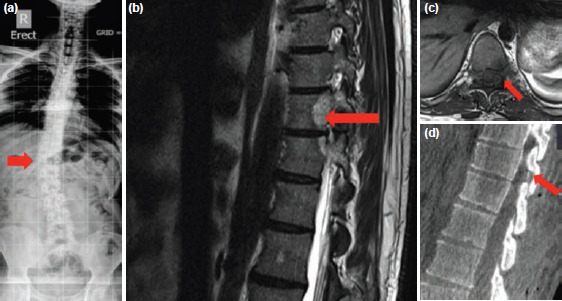
(a) Radiograph full length anteroposterior view showing the scoliosis with convexity to the right. (b) A well-defined lytic lesion seen on MRI sagittal cut involving the superior articular process of the D11 vertebra. (c) MRI axial cut showing lesion superior articular process of D11. (d) Intra-operative image showing adequacy of extirpation using the navigation probe in sagittal image.

A 33-year-old male presented with dull mid-back pain radiating to the left side of the chest for 7 months. Pain was aggravated at night and relieved by taking nonsteroidal anti-inflammatory drugs (NSAIDS). Pain progressively increased to the point of causing difficulty in daily activities. On clinical examination, he had dorsolumbar scoliosis with convexity to the right, tenderness, and paraspinal spasm in that region. The scoliosis was partially corrected on bending forward. His back visual analog scale (VAS) score was 8 with Oswestry Disability Index 40%. Neurological examination was normal. Radiographs of the dorsolumbar spine suggested sclerosis in the D10 and D11 facet joint on the left side with scoliosis with convexity to the right. Pre- and post-contrast magnetic resonance imaging of the dorsolumbar spine was performed. The dynamic evaluation was performed with contact using time-intensity curves and correlated with plain CT. There was a well-defined lytic lesion seen involving the superior articular process of the D11 vertebra measuring 11mm x 11mm x 9mm being hypointense on T2 and mildly hyperintense on T1 with central sclerosis on CT scan scalloping the inferior articular process of the D10 vertebra. It showed rapid early uptake of contrast on arterial phase with slow washout. These findings favoured a diagnosis of osteoid osteoma (Fig. 1).

### Case 2

**Fig 2:**
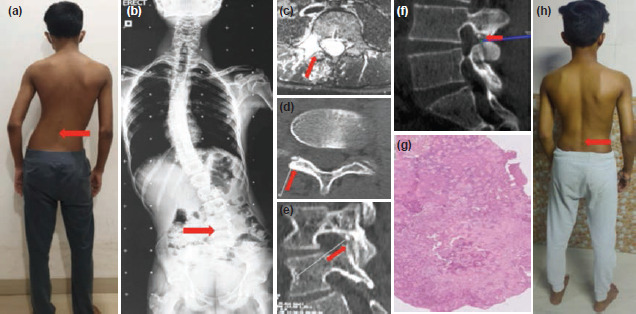
(a) Clinical examination shows lumbar scoliosis with convexity to left. (b) Radiograph full length anteroposterior view showing lumbar scoliosis with convexity to the left. (c) Lytic lesion with marrow edema and enhancement focus involving the L4 right pars interarticularis seen on MRI axial cut. (d) A well-defined nidus with radiolucency and central sclerosis seen on axial cut on CT scan. (e) A well-defined nidus seen at pars interarticularis on sagittal cut on CT scan. (f) Checking the adequacy of extirpation using the navigation probe. (g) Histopathological examination confirmed diagnosis of osteiod osteoma. (h) Six months follow-up clinical picture showing correction of lumbar scoliosis.

A 17-year-old male presented with low-back pain for eight months. Pain was aggravated at night and relieved by taking nonsteroidal anti-inflammatory drugs (NSAIDS). Pain progressively increased to the point of causing difficulty in daily activities. Clinical examination showed lumbar scoliosis with convexity to left, tenderness, and paraspinal spasm in that region. The scoliosis was partially corrected on bending forward. His back visual analog scale (VAS) score was 9 with Oswestry Disability Index 44%. Neurological examination was normal. Radiographs of the lumbar spine suggested sclerosis in the L4 right pars interarticularis and lumbar scoliosis with convexity to the left. Pre- and post-contrast magnetic resonance imaging of the lumbar spine was performed. The dynamic evaluation was performed with contact using time-intensity curves and correlated with plain CT. There was an area of well-defined radiolucency with central sclerotic focus seen involving the L4 right pars interarticularis measuring 4mm. It showed rapid early uptake of contrast on arterial phase with slow washout. These findings favoured a diagnosis of osteoid osteoma which was confirmed on histopathology (Fig. 2).

The patient was positioned prone on the Jackson table [Mizuho OSI, Tokyo, Japan]. After taking safe surgical precautions, a single two-dimensional fluoroscopy image was taken from the O-arm [Medtronic, Memphis, TN, USA] to mark the incision and a stab incision was made cranially to attach the reference probe to the spinous process, extending caudally to the dorsolumbar region. Subsequent dissection was performed to reach the left D10 D11 facet joint for first case. For the second case, the Medtronic METRx 14mm tube system was used with Wiltse approach. With O-arm setting changed to minimise the radiation dose; a CT scan was taken within 15s. The navigation system used was StealthStation S8 [Medtronic, TN, USA]. The basic data used for navigation consisted of pre-operative CT data, which were transferred and recorded on the system computer and reconstructed into three-dimensional (3D) images.

After registering the navigation with a 1.5mm ball-tipped reference probe, the accuracy of the images was verified by point merging and surface merging. The lesion was identified and accurately localised with the probe. Under neurosurgical microscope OPMI Pentero 900 [Zeiss, Oberkochen, Germany] careful drilling was initiated with 3mm diamond tip MIDAS Navidrill [Medtronic, TN, USA] till the lesion wall was reached as identified with the probe. The lesion was extirpated en masse in both cases. Further drilling was done in the walls of the articular process taking care not to breach the pedicle and keeping the dura safe.

Adequacy of extirpation was checked using the Navigation probe. Total amount of blood loss was less than 30ml in both cases. The operative time for the first case was 90min and the second case was 75min. Surgical wound was closed. Both patients walked 4h after the surgery. On the second day of surgery, the back VAS improved to 2, and patients were discharged home. Histopathological examination confirmed osteoid osteoma diagnosis. After follow-up of one year, both did not complain of back pain or radiation, scoliosis had improved in both cases VAS was 0 and ODI was 0%

## Discussion

Treatment of an osteoid osteoma involves complete intralesional excision of the nidus, either by surgical or radiological intervention after failure of adequate medical management. Although, intra-operative nidus localisation is challenging and can lead to extensive resection of the adjacent normal bone structure leading to risk of iatrogenic fracture and extended period of healing, instability requiring stabilisation, or neurovascular injury. Accuracy of intra-operative localisation of the nidus include radionuclide localisation with a gamma probe, intra-operative CT-guided localisation, or a combination of scintigraphy and computer navigation^2^. CT-guided percutaneous resection technique has limitations such as accurate localising the drill tip leading to malposition and inadequate drilling with increased risk of inadvertent dural and spinal cord injury, or increased risk of infection due to substandard sterility precautions in radiological suites. CT-guided percutaneous radiofrequency thermal ablation and laser photocoagulation for the treatment of osteoid osteomas located in the extremities has been used successfully. However, proximity to neural structures makes this modality conflicting in the spine. Thermal damage to the neural structures due to the heating tip has always raised concerns. Inadvertent injury to adjacent neural structures, high failure rates in the spine and lack of histologic confirmation of diagnosis makes it less ideal for treating osteoid osteomas in the spine.

Advantages of a three-dimensional (3D) computed tomography-based navigation system has recently been used for safe, accurate, reliable spinal tumour excision^3^. Gasbarrini *et al*^4^ studied 81 patients operated for osteoid osteoma either by conventional excision therapy or minimally invasive surgery (six of 81). They concluded that minimally invasive surgery has advantages of reducing soft tissue morbidity and the collateral damage caused by conventional surgical approach, it allows patients a more rapid and complete return to normal function. Considering accuracy of 3D fluoroscopic navigation, Mason *et al*^5^ analysed 30 studies for accuracy of pedicle screws. They reported accuracy of conventional fluoroscopy as 68.1%; accuracy of 2D fluoroscopic navigation as 84.3%; and with 3D fluoroscopic navigation accuracy as 95.5%. They concluded that accuracy rates of 3D as compared with 2D fluoroscopic navigation were consistently higher throughout all individual spinal levels. This result show that 3D fluoroscopic navigation can help surgical excision of osteoid osteoma more accurately.

Limitations of this study includes lack of long-term follow-up to evaluate for recurrence. Also, large group of patients is required to make this a standardised treatment for osteoid osteoma.

This study of 3D navigation with the O-arm system provided precise, safe, accurate, reliable localisation of the lesion, reducing approach related morbidity, the risk of iatrogenic instability and subsequent instrumented stabilisation, thereby reducing the hospital stay and cost of treatment. An intra-operative verification O-arm spin confirmed complete tumour excision and a low dose protocol of the O-arm significantly reduced the radiation exposure to the patient. Histopathological confirmation of the diagnosis can also be provided by this technique.
